# When is the use of pacifiers justifiable in the baby-friendly hospital initiative context? A clinician’s guide

**DOI:** 10.1186/s12884-017-1306-8

**Published:** 2017-04-27

**Authors:** Welma Lubbe, Wilma ten Ham-Baloyi

**Affiliations:** 1 North-West University, School of Nursing Science, Private Bag X6001, Potchefstroom, 2520 South Africa; 2 Nelson Mandela Metropolitan University, Faculty of Health Sciences, Private Bag 77000, Port Elizabeth, 6031 South Africa

**Keywords:** Breastfeeding, Pacifier, Baby-friendly hospital initiative, Clinician’s guide, Neonatal intensive care unit, Pacifier risks, Justifiable pacifier use, Recommendations for pacifier use

## Abstract

**Background:**

The use of pacifiers is an ancient practice, but often becomes a point of debate when parents and professionals aim to protect and promote breastfeeding as most appropriately for nurturing infants. We discuss the current literature available on pacifier use to enable critical decision-making regarding justifiable use of pacifiers, especially in the Baby-Friendly Hospital Initiative context, and we provide practical guidelines for clinicians.

**Discussion:**

Suck-swallow-breathe coordination is an important skill that every newborn must acquire for feeding success. In most cases the development and maintenance of the sucking reflex is not a problem, but sometimes the skill may be compromised due to factors such as mother–infant separation or medical conditions. In such situations the use of pacifiers can be considered therapeutic and even provide medical benefits to infants, including reducing the risk of sudden infant death syndrome. The argument opposing pacifier use, however, is based on potential risks such as nipple confusion and early cessation of breastfeeding. The Ten Steps to Successful Breastfeeding as embedded in the Baby-Friendly Hospital Initiative initially prohibited the use of pacifiers in a breastfeeding friendly environment to prevent potential associated risks. This article provides a summary of the evidence on the benefits of non-nutritive sucking, risks associated with pacifier use, an identification of the implications regarded as ‘justifiable’ in the clinical use of pacifiers and a comprehensive discussion to support the recommendations for safe pacifier use in healthy, full-term, and ill and preterm infants.

**Summary:**

The use of pacifiers is justifiable in certain situations and will support breastfeeding rather than interfere with it. Justifiable conditions have been identified as: low-birth weight and premature infants; infants at risk for hypoglyceamia; infants in need of oral stimulation to develop, maintain and mature the sucking reflex in preterm infants; and the achievement of neurobehavioural organisation. Medical benefits associated with the use of pacifiers include providing comfort, contributing towards neurobehavioural organisation, and reducing the risk of sudden infant death syndrome. Guidelines are presented for assessing and guiding safe pacifier use, for specific design to ensure safety, and for cessation of use to ensure normal childhood development.

## Background

Exclusive breastfeeding in newborn infants is well researched and agreed upon as an essential aspect of newborn care as is evident from guidelines by the World Health Organization (WHO). Sucking is an important milestone for every newborn infant to master to ensure exclusive breastfeeding after birth, and it also contributes to self-regulatory behaviour [1] and bonding. Non-nutritive sucking (NNS), or sucking not for the purpose of feeding, is a precursor to nutritive sucking [2, 3], and holds various physiological benefits including improved digestion, behavioural organisation [4], pain management, and prevention of aspiration [5]. Furthermore, the American Academy of Pediatric (AAP) has published recommendations on the use of pacifiers in healthy infants associated with a reduction in the risk of sudden infant death syndrome (SIDS) [6]. Although these benefits have been documented, the use of pacifiers to support NNS is not welcomed in pro-breastfeeding contexts, such as hospitals holding or working towards obtaining Baby Friendly Hospital Initiative (BFHI) status, and consequently also not welcomed in neonatal intensive care environments.

However, the risk of losing the sucking reflex has been identified in infants separated from their mothers for extended periods [4], and the contribution of NNS has been highlighted for preserving the reflex and enhancing physiological stability in infants, especially in situations of mother–infant separation when, for example, the infant is admitted to a neonatal intensive care unit (NICU) [7] or the mother is very ill and admitted to high or intensive care. Healthcare professionals have started to review the prohibited use of pacifiers as stated in the ‘Ten Steps to Successful Breastfeeding’ as part of the BFHI. The Nordic and Quebec working group [8], for example, has proposed expanding the BFHI Ten Steps to include the use of pacifiers in the NICU when justifiable as the current BFHI policy does not include the NICU context at all. Another approach to “Promoting and Protecting Breastfeeding for Vulnerable Infants” is the Spatz Ten Steps Model. This model outlines the following ten steps: providing parents with information to make an informed decision to breastfeed; assisting mothers with the establishment and maintenance of milk supply; correct breast milk management (storage and handling); developing procedures to feed the infant the breast milk; skin-to-skin care creates opportunities for NNS at the breast; managing the transition to breast; measuring milk transfer; preparation of infant and family for discharge; and appropriate follow-up care [9]. Implementation of this model in an 82-bedded NICU over a period of three years (2010–2013) resulted in an increased parent satisfaction with nurses’ support of breastfeeding and a 3.1‐fold greater odds of the infant receiving mother's own milk at discharge [10]. None of the available models however include the developing suck-swallow-breathe (SSB) co-ordination as the final step in transition to oral feeds.

To clarify the current points of departure and opinions regarding the use of pacifiers in ill and healthy infants, and to provide evidence supporting the use or non-use of pacifiers in cases of mother–infant separation, we present literature to support informed, clinical decision-making on the appropriate use of pacifiers in various situations. We discuss the literature dealing with the development of sucking during the fetal period; background to the BFHI; and the expansion of the Ten Steps as presented by Nyqvist et al [8]. We also discuss the value of NNS and presumed risks associated with pacifier use, and we conclude with recommendations for pacifier use as presented by Sexton and Natale [11] and the AAP [12]. Our paper adds to the knowledge base by providing arguments and evidence that allow clinicians to make informed decisions on appropriate circumstances for the use of pacifiers, and by outlining a scientific and evidence-based clinical guide for pacifier use.

## Discussion

### Fetal development of sucking

Sucking is an important part of feeding; this skill starts to develop in utero as early as the eighth week of gestation and continues well after birth [13]. NNS (sucking for purposes other than obtaining nutrition) precedes nutritive sucking and can be observed during the fetal period as early as 13 weeks post menstrual age (PMA) [2]. Sucking movements are observed in the fetus between the 24^th^ and 28^th^ week of gestation, and coordination of the suck, swallow and breathing pattern is evident from week 28 [14]. This pattern becomes a mature rhythm only after 36 weeks, however, and in some infants only after birth, depending on their individual level of maturity, which is vital in effective feeding from the breast.

Infants utilize reflexive responses associated with feeding, such as suck/swallow, tongue thrust, rooting and gag reflexes, which enable them to locate the food source and feed effectively [15]. If any of the reflexes are absent, feeding may be interrupted at birth. In addition, sucking may be delayed, interrupted, and even seen as a developmental challenge in immature and preterm infants, such as those admitted to an NICU, since the more immature the infant, the poorer the ability to suck [3]. Furthermore, as Arvedson reported in 2006, normal development of sucking and feeding is significant for understanding feeding disorders in infants and children [16]. Although reflexes are important; disorders in the SSB is the determining factor.

For the purpose of this debate it is important to explore the value of NNS for the healthy infant, the high risk infant, and the infant who is ill.

### The value of NNS

Specific medical benefits related to pacifier use indicate that the practice could be beneficial in NICUs for NNS and to comfort the preterm or sick infant [1]. NNS is the oral motor skill that precedes feeding [17]. It has a two-part distinctive developmental role: first, it supports the immature infant in developing mature and well-regulated sucking; second, it contributes to oral feeding by supporting physiological stability and multiple other benefits associated with behavioural, motor and neurological systems [18].

**Physiological benefits** such as increasing levels of oxygenation and decreased heart rate (especially important in the case of stressed infants with tachycardia) [4, 8] have been reported with NNS. NNS is further associated with improved glucose-utilization because sucking stimulates the vagus nerve, which causes somatostatin to decrease and gastrin secretion to rise. As a result the absorption of milk feeds is increased and digestion improved [8].

#### Behavioural benefits

Behavioural organization is improved in infants exposed to NNS, since the practice improves self-consolation and soothing in the infant [19], self-regulatory state modulation [4], and the time spent sleeping, and it increases levels of alertness [2]. All these contribute to lower energy consumption. Giving the infant five (5) minutes of NNS before a feed is, furthermore, associated with increased alertness during feeds, leading to greater feeding [20]. The treatment plan of infants struggling with poor suck, swallow and breathe coordination can also include NNS, since it aids in neurobehavioral organization and maturation [4]. Furthermore, in a crossover study including 30 preterm infants, a pacifier did not had an effect on acid and nonacid gastro-esophageal reflux, and may therefore be used in preterm neonates with gastro-esophageal reflux [21].

#### Motor system benefits

NNS contributes to improvement in muscle tone and coordination [16], which is an important aspect of energy conservation and growth.

#### Neurological benefits

Accelerated maturation and improved pain management can be classified as neurological benefits. The use of NNS in conjunction with a sweet substance, such as expressed breast milk or sucrose, for managing pain during painful procedures and interventions such as immunizations [19, 22], and heel pricks [23] are often underutilized, especially in preterm infants and full term infants up to six months [11]. In addition, the benefits of NNS specifically for immature preterm infants are even more far reaching. It protects the infant against aspiration, since suck inhibits swallowing, and in tube-fed infants it contributes to accelerated maturation, which in turn contributes to faster transition from tube to oral feeds [4, 24]. All these benefits lead to greater weight gain and, in the hospitalized infant, to earlier discharge [11, 19, 24].

#### SIDS

Evidence suggests that using pacifiers in older infants at nap times and bedtime at night is associated with a decrease in the risk of SIDS [1, 19, 22, 25, 26]. The reduced risk was, however, found in combination with other factors, such as mothers who were older, married, non-smoking, and breastfeeding, and mothers who had received adequate prenatal care [27].

However, with regards to the benefits outlined, mention should be made of the broad range of ages, feeding practices, deficits, and medical conditions in the cohorts of infants used in the above mentioned studies. Benefits of using pacifiers in the NICU’s can therefore not be established which leads to general recommendations for safe pacifier use, which will be outlined later in this paper.

### Techniques to support NNS

Different techniques can be used to support sucking, some of which are regarded as better as and safer than others. They include sucking on the infant’s own hands or fingers, on the mother’s ‘emptied’ breast [28], on an adult’s finger, or even on an oro-gastric tube in the case of a hospitalised infant [29], as well as sucking on pacifiers.

The use of pacifiers (also referred to as dummies, soothers or artificial teats) for sucking dates back thousands of years [30] and is helpful when the infant’s self-regulation needs support but when the mother is unavailable to provide comfort and sucking on the breast, and when alternative measures to support self-regulation are insufficient.

In 2009, UNICEF described oral feeding as an art and a science and not something that happens automatically. It has become increasingly clear that additional support may be required for infant feeding and, as indicated in a joint WHO/UNICEF statement as long ago as 1989; such support ought to be provided by trained professionals from a multidisciplinary team [31].

### The BHFI context

Prior to 1990 breastfeeding rates were declining globally. As a result the Baby Friendly Hospital Initiative (BFHI) was launched in 1990 by James Grant, Executive Director of UNICEF, and Hiroshi Nakajima, Director-General of WHO, with the aims of transforming healthcare policies by protecting and promoting breastfeeding through restoring it as the natural and normal practice for nurturing babies; providing mothers and babies with a good start for breastfeeding; and increasing the likelihood that babies would be breastfed exclusively for the first six months and then given appropriate complementary foods while continuing with breastfeeding for two years or beyond [31, 32]. Furthermore, breastfeeding was identified as an essential part of newborn care to improve infant survival and reach the Millennium Development Goals, and currently the Sustainable Development Goals. The use of human milk, whether this is through breastfeeding or expressing breastmilk has shown multiple benefits, specifically for those infants that are admitted to NICU. Human milk is regarded as the safest feeding option as it improves among other things, the infant’s neurocognitive and developmental outcomes as well as it assists in developing the infant’s immune system and enables the mother to tailor the milk to the infant’s needs. Mothers should thus be made aware about the benefits of using human milk to feed their infants that are admitted to NICU. Being able to provide their infant in NICU with human milk can be perceived as a rewarding activity during which mothers feel they can be part of their infant’s healing [33]. Furthermore, the mothers should be encouraged to use human milk and receive the support to decide on the most suitable feeding practice for them, set their personal feeding goals and they can be taught how to express breastmilk, how to do mouth care with human milk, skin-to-skin care, NNS sucking at the breast, as well as transitioning to breast feeds [34–36]. A Breastfeeding Resource Nurse (BRN) could play an important role in the support of mothers and neonatal staff with regards to breastfeeding and expressing breastmilk [37–39].

#### Expansion of the Ten steps

The importance of the BFHI and the value of exclusive breastfeeding in full-term and ill neonates are well recognized. However, implementing the ‘Ten Steps to Successful Breastfeeding’ proved challenging in the ill and high risk neonatal environment where mother and baby are often separated from each other. As a result, in March 2009 in Copenhagen, the Nordic and Quebec working group was formed comprising health professionals from Sweden, Norway, Denmark, Finland and Quebec, Canada, to address expansion of the BFHI to neonatal care. In 2013 they published the proposed document: *Neo-BFHI: The Baby Friendly Hospital Initiative for Neonatal Wards. Three Guiding Principles and Ten Steps to protect, promote and support breastfeeding. Core document with recommended standards and criteria* [8]. This document addresses all the Ten Steps in the original WHO guideline but, in addition, makes provision for the special circumstances in the NICU. Table 1 presents the original Ten Steps, highlights the proposed adaptations for the NICU environment and show the alignment thereof with Spatz’s model for protecting breastfeeding for vulnerable infants.

**Table 1:** Comparison of the original Ten Steps to Successful Breastfeeding, the proposed expanded steps to successful breastfeeding and Spatz’s Ten Steps for Promoting and Protecting Breastfeeding for Vulnerable Infants

Original Ten Steps to Successful Breastfeeding [40] Every facility providing maternity services and care for newborns should implement the following Ten Steps	Expanded BFHI for Neonatal Units [8]	Promoting and Protecting Breastfeeding for Vulnerable Infants in the Spatz Ten Steps Model [9]
1. Have a written breastfeeding policy that is routinely communicated to all health care staff.	No change.	
2. Train all health care staff in skills necessary to implement this policy.	Educate and train all staff in the specific knowledge and skills necessary to implement this policy.	Correct breast milk management (storage and handling)
3. Inform all pregnant women about the benefits and management of breastfeeding.	Inform all hospitalized pregnant women at risk for preterm delivery or birth of a sick infant about the management of lactation and breastfeeding and benefits of breastfeeding.	Providing parents with information to make an informed decision to breastfeed
4. Help mothers initiate breastfeeding within a half-hour of birth.	Encourage early, continuous, and prolonged mother–infant skin-to-skin contact (kangaroo mother care) without unjustified restrictions. Place babies in skin-to-skin contact with their mothers immediately following birth for at least an hour. Encourage mothers to recognize when their babies are ready to breastfeed and offer help if needed.	Assisting mother with the establishment and maintenance of milk supply. Skin-to-skin care Create opportunities for NNS at the breast
5. Show mothers how to maintain lactation even if they are separated from their infants.	Show mothers how to initiate and maintain lactation and establish early breastfeeding with infant stability as the only criterion.	Managing the transition to breast
6. Give newborns no food or drink other than breastmilk unless medically indicated.	No change.	Develop procedures to feed the infant the breast milk
7. Practise rooming-in – that is, allow mothers and infants to remain together 24 h a day.	Enable mothers and infants to remain together 24 h a day.	
8. Encourage breastfeeding on demand.	Encourage demand feeding or, when needed, semi-demand feeding as a transitional strategy for preterm and sick infants.	Measuring milk transfer
9. Give no artificial teats (also called dummies or soothers) to breastfeeding infants.	Use alternatives to bottle-feeding at least until breastfeeding is well established and use pacifiers and nipple shields only for justifiable reasons.	
10. Foster the establishment of breastfeeding support groups and refer mothers to them on discharge from the hospital or clinic.	Prepare parents for continued breastfeeding and ensure access to support services/groups after hospital discharge.	Preparation of infant and family for discharge; and appropriate follow-up care

The literature is clear that pacifiers are essential and beneficial in certain situations, but several risk factors are often presented in arguments against their use. The presumed risks are outlined as follows.

### Presumed risks associated with pacifier use

Resistance to the use of pacifiers according to the Original Ten Steps to Successful Breastfeeding [40] (as outlined in Table 1, Step 9) in a breastfeeding supportive context is based on presumed risk factors for which, however, little supporting evidence can be found, and whose relevance to the young infant population has not been established.

Potential risks of pacifier use were highlighted in Sexton and Natale’s [11] review for different age groups. For example, in the full term and older infant up to six months, early breast weaning can be a complication of pacifier use and, when the use of pacifiers is prolonged, risks include otitis media (six months to two years) and dental malocclusion, for example, misalignment of the teeth such as open bite, cross bite or over jet (two years and older) [11]. Castilho and Rocha [30] suggest that the use of pacifiers may cause suffocation, poisoning, or allergies, and increase the risk of caries, infections, and intestinal parasitic diseases. Pacifier use was said to cause nipple confusion in breastfeeding infants, however no physical evidence validating such a confusion could be found in the literature, and infants seem to be able to differentiate between nutritive and non-nutritive sucking [41]. Although one large randomized controlled trial including 700 breastfed newborns found that pacifier use shortened breastfeeding duration, it did not affect exclusive or full duration of breastfeeding [42]. Furthermore, recent evidence suggests that pacifier use is a marker of breastfeeding difficulties or reduced motivation to breastfeed, rather than the cause of early weaning [41, 43].

Use of a pacifier should be restricted for infants with chronic or recurrent otitis media [1, 22, 44, 45]. Rovers et al [45] explain that the pacifier induces reflux of nasopharyngeal secretions into the middle ear, which may in turn increase susceptibility to acute otitis media. The first middle ear infection causes damage to the mucosa of the middle ear, thereby predisposing the infant to further infection. Sexton and Natale therefore recommend the use of pacifiers only for the first six months and thereafter weaning is recommended in order to avoid the general risks as outlined in this section [11].

### Implications for clinical practice

In line with Step 9 of the Neo-BFHI document, which states that ‘pacifiers should be used only for justifiable reasons’ [8], we propose the use of pacifiers as a useful aid, for preterm or ill infants, only when the situation calls for it and when the medical conditions listed below are met. We will also provide a summary of justifiable pacifier use in full-term infants, which is mostly in the out-of-hospital context.

#### Medical conditions justifying pacifier use

Nyqvist et al [8] suggested the provision of pacifier sucking in the hospital when ‘justifiable’. It may be beneficial to use pacifiers in the hospital setting in the circumstances summarized below, but it is extremely important for all possible effort to be made to prevent mother–infant separation, and to consider pacifier use only when the dyad cannot be kept together. It is thus important to consider the mothers own emptied breast, where possible, as a pacifier for NNS [28]. Justifiable medical conditions for the use of pacifiers, when the mother is not able to provide sucking on the empty breast, include the following:

- Infants weighing less than 1,500 g and/or is younger than 32 weeks gestational age [31];
- Infants at risk for hypoglycaemia [31];
- Infants in need of early oral stimulation to maintain and develop the sucking reflex [4];
- Severe illness of the mother preventing her from breastfeeding (temporarily or permanently), such as Herpes simplex virus type 1 [31];
- Maternal medication preventing mother from breastfeeding, such as sedating psychotherapeutic drugs and cytotoxic chemotherapy [31];
- Infants in NICU environments in need of calming, pain relief and decrease of stress [8];
- Infants receiving tube feeds [8].

The criteria for justifiable use has been provided in this section, however due to the broad range of ages, feeding practices, deficits, and medical conditions in the cohorts of infants used in the studies benefits of using pacifiers in the NICU’s could therefore not be established which lead to general recommendations for safe pacifier use, as outlined in the following section.

#### General recommendations for safe pacifier use

*General* recommendations for determining the appropriateness of pacifier use for healthy and preterm infants include determining their individual feeding programme by qualified health professionals [31]. Parents and caregivers should routinely be counselled by such professionals about safe and appropriate pacifier use [1, 12, 44], and information given to parents should include justifiable reasons for pacifier use in hospital [8], as well as alternative ways of soothing the infant, such as using the mother’s emptied breast [28], and recommendations to minimize pacifier use [8].

##### Delayed introduction and limited use

The introduction of a pacifier in full term infants should be delayed until one month of age to ensure the establishment of breastfeeding [12, 19, 46], and thereafter its use should be limited to the soothing of a breast-fed infant [22, 44]. It is therefore important for parents to be able to differentiate between a hungry baby, who will continue crying when the pacifier is removed from the mouth, and an infant who needs comforting through sucking. Pacifiers should never be used to replace or delay meals with full term infants, and should be offered only when the caregiver is certain that the child is not hungry [12].

#### Combined use

Pacifiers in combination with the mother’s voice through using a pacifier-activated music player during NNS has shown some benefits in oral feeding skills in preterm infants and may be used, specifically in the NICU when the mother cannot use her breast as a pacifier [47].

Pacifiers should not be coated in any sweet solution [12] except when the pacifier and sucrose solution are used simultaneously for the purpose of pain management [22], for example in minor procedures in which glucose and pacifiers was found analgesic for preterm infants [48]. This sweet solution should preferably be mother’s own milk.

#### Sleep

The pacifier should be used when putting the infant down for sleep and it should not be reinserted after the infant falls asleep [12]. Parents and caregivers should be advised to exercise judgement and to restrain pacifier use. They should be taught to avoid ad lib use throughout the day [44].

Furthermore, in all cases, the health care professionals should recognize the use of a pacifier as a parental choice, which is determined by the needs of their infant [1].

#### Infection

To avoid infection, pacifiers should be cleaned often and replaced regularly [12]. They should never be shared among siblings and never licked to clean them. Parents should consider having several pacifiers available so as to rotate them through cycles of cleaning and use during the day [44].

In a happy infant, a pacifier should be used for pacifying, to support sleep and self-regulation and not as a plug. Furthermore, bigger children should rather not be using a pacifier when playing and walking around, and do not need the pacifier to support with soothing [49].

#### Design safety

A pacifier should be a single-piece unit and made of durable material to prevent parts from dislodging and posing a choking hazard. It should be replaced when worn, and never tied with a string to the crib or around a child’s neck or hand [12, 44]. A symmetrical nipple shape allows the pacifier to remain in the correct sucking position [22]. Flanges should have minimum horizontal and vertical dimensions of 43 mm to prevent the pacifier from lodging in the soft palate, and manufacturers should be required to place a ring behind the flange for removal in case of aspiration [22, 44]. Pacifiers should have a large shield that is wider than the child’s mouth (just over 3 cm in diameter) [12, 44].

Shields should have ventilation holes, which are essential to permit air passage [12, 22, 44], with a textured inner surface to help to prevent irritation and rashes that result from trapped saliva [22].

#### Cessation of pacifier use

Pacifier use should not be actively discouraged and may be especially beneficial in the first six months of life. The American Academy of Pediatrics and the American Academy of Family Physicians recommend weaning children from pacifiers in the second six months of life to prevent otitis media and Sexton and Natale support this recommendation, in support of avoiding dental problems [11].

Cessation of pacifier use is recommended by various authors. Although they vary regarding cessation age, they agree that curtailing should start at the age of three years and that the habit should be discontinued by or before the age of four years to minimize the development of malocclusion [22, 44]. From a speech therapy perspective, clinicians recommend cessation at 14 months of age to prevent interference with speech. Since these recommendations seem to be conflicting we suggest weaning from as early as six months and if the situation requires, not later than four years of age.

#### Recommendations for future research

It was evident from the review of the available literature that there are many different aspects which influence the safe use of pacifiers in both the full term and ill or preterm infant populations. Furthermore, it was clear from the literature that even within the breastfeeding and baby-friendly hospital environment there is a need for the use of pacifier is specific circumstances. These circumstances have been identified, however studies exploring the benefits of pacifier use in different age ranges, feeding practices, presence of deficits and medical conditions with clear guidelines for implementation were not documented in the literature. Systematic reviews have been done to determine the benefits of NNS, but can be expanded by exploring benefits specific to specific age groups and medical conditions. Limited research is available on the best design of pacifiers, and whether this is the best method of providing NNS. Research on design of pacifiers for both the preterm and fullterm infants can thus be useful. Finally, risks associated with pacifier use should further be explored and identified for various age groups, levels of illness and other differentiating circumstances Table 2.

**Table 2:** Summary of benefits, risks, implications and recommendations for safe pacifier use

Benefits of NNS	Risks associated with pacifier use	Implications for clinical practice – ‘justifiable use’	Recommendations for safe pacifier use
Physiological benefits • Increased levels of oxygenation • Decreased heart rate • Improved glucose-utilization resulting in increased improved digestion. Gastro-intestinal: • Does not affect acid and non-acid gastro-oesophageal reflux	Full term up to six months: May result in: • Early breast weaning • Otitis media • Dental malocclusion • Suffocation • Poisoning • Allergies • Increased risk of caries • Infections • Intestinal parasitic disease • Nipple confusion (not proven) • Shortened breastfeeding duration	Medical conditions • < 1,500 g and/or < 32 weeks gestation • At risk for hypoglycaemia • Needing oral stimulation to maintain and develop sucking reflex • Severe maternal illness preventing breastfeeding (e.g. Herpes Simplex) • Maternal medication contra-indicated for breastfeeding (e.g. psychotherapeutic drugs) • NICU infant needing calming, pain relief and stress management • During tube feedings	General: • Determine individual feeding programme by qualified health professional • Counsel parents and caregivers about safe and appropriate pacifier use • Information provided should include ‘justifiable’ reasons for pacifier use in hospital • Information should include alternative ways of infant soothing • Recommendations to minimize pacifier use should be provided. Delay introduction and limited use: • Delay introduction of use until one month of age to establish breastfeeding • Limit use to soothing of a breast-fed infant • Parents to differentiate between a hungry baby or in need of comforting by means of sucking • Not used to delay or replace meals
Behavioural: • Self-consolation and soothing • Self-regulatory state modulation • Comforts sick/preterm infant • Increased time sleeping • Increased alertness with better feeding • Lower energy consumption			Combination use: • Combine pacifier use with maternal voice • Do not coat pacifier in sweet solution, except when used simultaneously for pain relief Sleep: • Use when putting down to sleep and do not re-insert when infant falls asleep. • Avoid ad lib use throughout the day • Do not use to replace or delay meals in full term infants • Pacifier use is a parental choice Infection: • Avoid infection by cleaning and replacing pacifier regularly – do not lick • Never share between siblings • Bigger children should not play or walk around with a pacifier.
Motor system: • Improved muscle tone and coordination			Cessation: • Weaning from six months of age to prevent otitis media and dental problems • Start cessation at age six months and if situation requires no later than four years of age.
Neurological: • Precedes nutritive feeding by supporting accelerated maturation of sucking • Aids neurobehavioral organization and coordination in poor suck, swallow and breathe coordination • Protects against aspiration • Faster transition to oral feeds • Pain management • Better weight gain • Earlier discharge			Design safety: • Use a single-piece unit only • Made of durable material to prevent choking hazard • Replace when worn out • Never tie a string to the pacifier to prevent strangling the child • Symmetrical nipple shape to support correct tongue position when sucking • Flanges minimum dimensions of 43 mm to prevent lodging in the soft palate • Ring behind the flange for removal in case of aspiration • Mouth shield larger than the infants mouth (over 3 cm) • Ventilation holes in shields to permit air passage • Texture inner surface to prevent irritation and rashes from trapped saliva
SIDS: • Used at bedtime reduce risk for SIDS			

## Conclusions

Breastfeeding or the use of expressed human milk is an essential practice that should be protected and promoted to ensure the survival of newborn infants. The practice of breastfeeding has moved through different phases of popularity over the decades, but currently the evidence is overwhelmingly in support of it. Its importance should be highlighted and its practice should be supported by all healthcare professionals.

The drive towards best practice gave rise to initiatives such as the Baby-Friendly Hospital Initiative, which includes Ten Steps to Successful Breastfeeding. However, until recently this initiative did not provide for infants outside the healthy birth environment, such as high-risk, sick and immature infants separated from their mothers. In these circumstances additional support is required to achieve breastfeeding, and the literature strongly supports the use of pacifiers in justifiable situations to prevent delays in feeding development and to support infants in reaching maturity on different levels of development, such as feeding, motor organisation, neurobehaviour and self-regulation. Despite the evidence supporting the use of pacifiers, the practice encounters challenges because views vary on the matter. Furthermore, studies conducted vary in ages, feeding practices, deficits, and medical conditions in the cohorts of infants used which makes it difficult to draw definite conclusions regarding the safe use, duration and design of pacifiers in NICUs.

We have presented the argument that pacifier use is justifiable in specific conditions, such as immaturity and other medical situations and when the mother is not available. The benefits of using pacifiers are reported in the literature as supporting soothing in infants; enhancing maturation and protecting the suck reflex; and lowering the risk of SIDS. It is, however, essential to have guidelines available for clinicians to be able to assess the safety of a pacifier’s design and to be able to apply general measures to ensure safety when using pacifiers, specifically in the NICU context and/or with infants with special needs. Finally, pacifiers have an ‘expiry date’ and use should be ceased after six months of age to prevent complications such as otitis media and dental malocclusions.

A pacifier is an aid that can be used during mother–infant separation and to support breastfeeding in a therapeutic manner. Pacifiers should only be used in justifiable situations and in collaboration with or under the supervision of a healthcare professional who is concerned about protecting breastfeeding.

## Abbreviations

BFHI: Baby friendly hospital initiative
NICU: Neonatal intensive care unit
NNS: Non-nutritive sucking
SIDS: Sudden infant death syndrome
SSB: Suck-swallow-breathe
UNICEF: United Nations International Children’s Emergency Fund
WHO: World Health Organization
